# Probiotic treatment with specific lactobacilli does not improve an unfavorable vaginal microbiota prior to fertility treatment—A randomized, double-blinded, placebo-controlled trial

**DOI:** 10.3389/fendo.2022.1057022

**Published:** 2022-12-01

**Authors:** Ida E. Jepsen, Malene Hviid Saxtorph, Anne Lis Mikkelsen Englund, Kathrine Birch Petersen, Marie Louise Muff Wissing, Thomas Vauvert F. Hviid, Nicholas Macklon

**Affiliations:** ^1^ The Fertility Clinic, Department of Obstetrics and Gynecology, Zealand University Hospital, Koege, Denmark; ^2^ ReproHealth Research Consortium, Zealand University Hospital, Roskilde, Denmark; ^3^ Department of Clinical Medicine, University of Copenhagen, Copenhagen, Denmark; ^4^ TFP Stork Fertility, The Fertility Partnership Denmark, Copenhagen, Denmark; ^5^ Aleris-Hamlet Fertility, Aleris-Hamlet Hospital, Soeborg, Denmark; ^6^ Department of Clinical Biochemistry, Centre for Immune Regulation and Reproductive Immunology, Zealand University Hospital, Roskilde, Denmark; ^7^ London Women’s Clinic, London, United Kingdom

**Keywords:** microbiota, microbiome, vagina, probiotics, lactobacilli, IVF, randomized trial

## Abstract

**Objective:**

To investigate whether treatment with proprietary lactobacilli-loaded vaginal capsules improves an unfavorable vaginal microbiome diagnosed using a commercially available test and algorithm.

**Design:**

A randomized, double-blinded, placebo-controlled study was conducted in 74 women prior to undergoing fertility treatment at a single university fertility clinic between April 2019 and February 2021. The women were randomly assigned in a 1:1 ratio to receive one vaginal capsule per day for 10 days containing either a culture of more than 10^8^ CFU of *Lactobacillus gasseri* and more than 10^8^ CFU *Lactobacillus rhamnosus* (lactobacilli group) *or* no active ingredient (placebo group). Vaginal swabs for microbiota analysis were taken at enrollment, after treatment and in the cycle following treatment.

**Participants and methods:**

Women aged 18–40 years who prior to fertility treatment were diagnosed with an unfavorable vaginal microbiota, characterized by either a low relative load of *Lactobacillus* or a high proportion of disrupting bacteria using the criteria of the IS-pro™ diagnostic system (ARTPred, Amsterdam, the Netherlands), were enrolled in the study. The primary outcome measure was the proportion of women with improvement of the vaginal microbiota after intervention.

**Results:**

The vaginal microbiota improved after intervention in 34.2% of all participants (lactobacilli group 28.9%, placebo group 40.0%), with no significant difference in the improvement rate between the lactobacilli and placebo groups, RR = 0.72 (95% CI 0.38–1.38).

**Conclusion:**

This study indicates that administering vaginal probiotics may not be an effective means of modulating the vaginal microbiome for clinical purposes in an infertile population. However, a spontaneous improvement rate of 34.2% over a period of one to three months, confirming the dynamic nature of the vaginal microbiota, indicates that a strategy of postponing further IVF treatment to await microbiota improvement may be relevant in some patients, but further research is needed.

**Clinical trial registration:**

ClinicalTrials.gov, identifier NCT03843112.

## Introduction

The majority of *in vitro* fertilization (IVF) treatment cycles do not result in a pregnancy. While efforts to improve pregnancy rates remain largely focused on enhancing the quality of the transferred embryo, there is increasing awareness of the potential role of the intra-uterine environment as a determinant for success. Tests designed to assess different components of endometrial function are becoming commercially available (1) and with microbiota being shown to modulate organ function throughout the body, interest is growing in assessing the vaginal microbiota as a potentially treatable marker of endometrial receptivity and predictor of treatment outcome (2–4).

The term ‘microbiota’ describes the collection of microorganisms on or in the human body, where the term ‘microbiome’ describes the collection of microbes and their genomes (5). The healthy vaginal microbiota is dominated by *Lactobacillus* spp. with low microbial diversity compared to other body sites (6). High concentrations of estrogen are associated with the dominance of *Lactobacillus* spp (2, 3, 7). and its production of lactic acid results in a vaginal pH < 4.5, which in turn inhibits the growth of other bacteria and pathogens. The normal vaginal microbiota is dynamic and may vary with sexual activity and the phase of the menstrual cycle but appears stable between ovulation and the mid-luteal phase (8). At least five major community state types (CSTs) of vaginal microbiota can be distinguished in healthy and asymptomatic women based on two criteria: the relative abundance of *Lactobacillus* (*L.*) and the specific lactobacilli present (9). Four groups are relatively dominated by either *L. crispatus* (CST I), *L. gasseri* (CST II), *L. iners* (CST III), *or L. jensenii* (CST V), whereas the fifth (CST IV) is more heterogeneous and has a higher proportion of strictly anaerobic organisms (9). Dysbiosis, defined as a change from the normal vaginal microbiota homeostasis, has been reported to influence gynecological health and obstetric outcomes (2). The degree of dysbiosis can range from an asymptomatic state with a suboptimal bacterial composition to symptomatic bacterial vaginosis, increasing the risk of miscarriage and preterm birth in severe cases (10–13).

A number of studies have shown the vaginal microbiota to be predictive of the outcome of fertility treatments and suggest that dysbiosis in the reproductive tract negatively affects the chance of pregnancy following IVF treatment through yet unknown mechanisms (3, 4, 14–16). In a recent prospective study, an algorithm derived from vaginal microbiota profiling using IS-pro, a validated PCR based profiling technique, reported pregnancy rates after IVF treatment of 5.9%, 23.8%, and 52.5% in women with a microbiota reported as low, medium, or high favorability for implantation respectively (17).

The identification of the vaginal microbiota as a predictor for the chance of pregnancy following IVF suggests that optimization of the vaginal microbiota before IVF treatment might improve the chance of successful implantation. To address this, there is a need to identify interventions that can improve the putatively unfavorable vaginal microbiota. A range of strategies have been proposed ranging from simple dietary modifications to antibiotics and vaginal microbiota transplants (18). Particular interest exists in the potential of probiotic supplements, which are formulations containing live bacteria (19). Vaginal probiotic supplements containing live lactobacilli are available in different formulations over-the-counter. Although evidence is limited, probiotics may have beneficial effects in restoring normal vaginal colonization in bacterial vaginosis (20–23). However, it is unclear whether lactobacilli-loaded vaginal capsules represent an effective means of improving the vaginal microbiota diagnosed as of low implantation potential. The need for randomized controlled trials designed to elucidate this has recently been highlighted (24).

This study aimed to determine if lactobacilli-loaded vaginal capsules are superior to placebo in improving a vaginal microbiota reported as unfavorable to implantation in women scheduled for fertility treatment.

## Materials and methods

### Study design and approvals

A single-center, two-arm, double-blinded, randomized controlled study was conducted between April 2019 and February 2021 at the Zealand University Hospital Fertility Clinic, Denmark. The study was approved by the Danish National Committee on Health Research Ethics (SJ-710) and registered on the Clinical Trial website (www.ClinicalTrials.gov, identifier NCT03843112).

The study was monitored by an external monitor from the Danish Good Clinical Practice (GCP) center and reported in compliance with the CONSORT 2010 statement (25). All participants provided written informed consent. Adverse events and reactions were monitored systematically throughout the study.

### Study population

Women aged 18–40 years referred to the Fertility Clinic who prior to fertility treatment had been diagnosed with an unfavorable vaginal microbiota were invited to participate in the study.

All the participants were primary infertility patients referred for their first fertility treatment.

An unfavorable microbiota was defined using the criteria for ‘low’ and ‘medium’ profiles reported to be predictive of a poor or suboptimal chance of pregnancy following IVF as defined below (17).

Exclusion criteria were pregnancy, smoking, use of oral antibiotics between vaginal swab and inclusion, use of oral or vaginal probiotics between vaginal swab and inclusion, and allergy towards any ingredient in the vaginal capsules.

### Sample analysis

Samples for microbiota analysis were taken from the posterior vaginal fornix using designed-for-purpose swabs during a gynecological examination using sterile instruments and sterile saline water. Samples were sent for analysis using a next-generation sequencing-based IS-pro™ diagnostic system (ARTPred, Amsterdam, the Netherlands). The IS-pro method is a validated technique (26–30) which is described elsewhere (31). The overall result of the analysis placed the sample in one of three categories (‘low’, ‘medium’, or ‘high’ profile) using the criteria which Koedooder et al. found to be predictive of the pregnancy chance following IVF (17). The ‘low’ profile was characterized by a ‘*relative Lactobacillus (L.) load < 20%, relative load of L. jensenii > 35%, presence of Gardnerella vaginalis IST1 or Proteobacteria > 28% of total bacterial load*’. If none of these criteria was fulfilled, the sample was identified as either ‘medium’ profile or ‘high’ profile based on the relative abundance of *Lactobacillus crispatus* (medium ≥ 60%; high < 60%). Koedooder et al. found the chance of pregnancy within two months for IVF patients with low, medium, and high profiles to be 5.9%, 23.8%, and 52.6%, respectively. In addition to the overall profile, the analysis specified the composition of the sample microbiota by the relative presence of *Lactobacillus crispatus*, *Lactobacillus iners*, *Lactobacillus jensenii, Lactobacillus* spp., *Proteobacteria*, *Gardnerella vaginalis IST1*, and a residual group *other.*

### Study drug and placebo

The intervention consisted of vaginal capsules containing more than 10^8^ CFU of *Lactobacillus gasseri* EB01 DSM14869 and more than 10^8^ CFU *Lactobacillus rhamnosus* PB01 DSM14870. *Lactobacillus gasseri* is the dominant lactobacilli in healthy and asymptomatic women with community state type II (9). The strains are proprietary to Deerland Probiotics & Enzymes A/S, Hundested, Denmark and the capsules are marketed under the brand Vivag Plus^®^ by Orkla Care A/S, Hvidovre, Denmark. Other capsule ingredients include lactitol monohydrate, corn starch, gelatine, xanthan gum, glucose, magnesium stearate, and titanium dioxide (E171). The preparation has no reported side effects or adverse reactions. The placebo formulation was identical in content, appearance, and texture to the active study drug but devoid of bacterial strains. The manufacturer produced both the active study drug and the placebo formulation in identical packages containing 10 vaginal capsules according to good manufacturing practice (GMP). Participants were asked to return the empty packages as well as any excess medicine at their first follow-up visit. Study drugs were handled according to GMP.

### Randomization and blinding

Included women were randomized in a 1:1 ratio to either lactobacilli-loaded vaginal capsules or placebo. The pharmacy of the Capital Region of Denmark conducted the randomization using a computer-based randomization program (www.randomization.com) and labeled all drug packages to ensure blinding of both clinicians and participants to the content of the packages. Unblinding was not carried out until after completion of the study when all data had been entered and the statistical analysis plan confirmed.

### Study procedures and timing of samples

Baseline diagnostic samples to assess eligibility were taken upon consent on the day of first visit unless in case of current menstrual bleeding. Eligible participants were enrolled at the beginning of a menstruation cycle no later than two months after the baseline sample diagnosing an unfavorable microbiota. Data on menstrual cycle, BMI, and sexual history were collected.

Following randomization, labeled study medicine was handed out, and participants were instructed to take one vaginal capsule every night for 10 days starting on the first day after menstrual bleeding stopped, corresponding to CD 4–7. On CD 21–25 in the same cycle, a vaginal swab for microbiome analysis was taken (first sample after intervention). In the next cycle, a further vaginal swab was taken on CD 21–25 (second sample after intervention). Participants were advised not to undergo fertility treatment during the study period to avoid any influence from the hormonal effects of fertility treatment on the vaginal microbiota. Similarly, participants were instructed to refrain from the use of any probiotics during the study period and compliance was ensured at each visit by an interview.

### Outcome measures

The primary outcome measure was dichotomous: improvement or no improvement in the vaginal microbiota profile occurring in the period between the baseline sample and the first sample after the intervention. An improvement was defined as a shift in profile from low to medium, medium to high, or low to high, whereas no improvement was defined as no change, high to medium, or medium to low. To explore the persistency of any changes, we defined a secondary dichotomous outcome as improvement or no improvement in the vaginal microbiota profile between the baseline sample and the second sample after the intervention (collected in the subsequent menstrual cycle).

*Post hoc* exploratory analyses included quantification of the conversion rate overall in both groups, including the proportion of women who deteriorated to a less favorable microbiota profile between samples. The specific microbiota composition before and after intervention was analyzed by the average relative abundance of specific bacteria in the two groups. Further, the number of spontaneous pregnancies during the study period and the impact of sexual activity were quantified.

### Sample size/power calculation

The sample size was derived from a power calculation based on a number of assumptions, as little data was available to guide this. The premise was that 80% of women in the intervention group and 20% of women in the placebo group would demonstrate an improved vaginal microbiota profile in the intervention cycle. The rationale for this reflected consideration of the likely impact of other non-intervention modulating factors on the microbiota and an expectation of a spontaneous modulation rate before retesting in the next cycle of 20% in both groups. These yielded a proposed persistent improvement of the vaginal microbiota in 64% vs. 32% of women in the intervention and control group, respectively. With chosen parameters for power (1-β) = 0.80 and p-value (α) = 0.05, these assumptions necessitated 37 participants in each arm (1:1 ratio). The protocol allowed for replacement of participants dropping out before the first sample (primary outcome).

### Statistical analysis

The relative risk (chance) of improvement in the vaginal microbiota in the lactobacillus group compared to the placebo group was calculated from cross-tabulations for primary and secondary outcomes. *P*-values were calculated using Pearson’s chi-squared test. All data were collected and stored in a trial database (EasyTrial ApS, Aalborg, Denmark). Data were analyzed using SPSS version 27 (IBM, New York, USA). The statistical significance level was set at *P* < 0.05.

## Results

### Study participants

**Figure 1:**
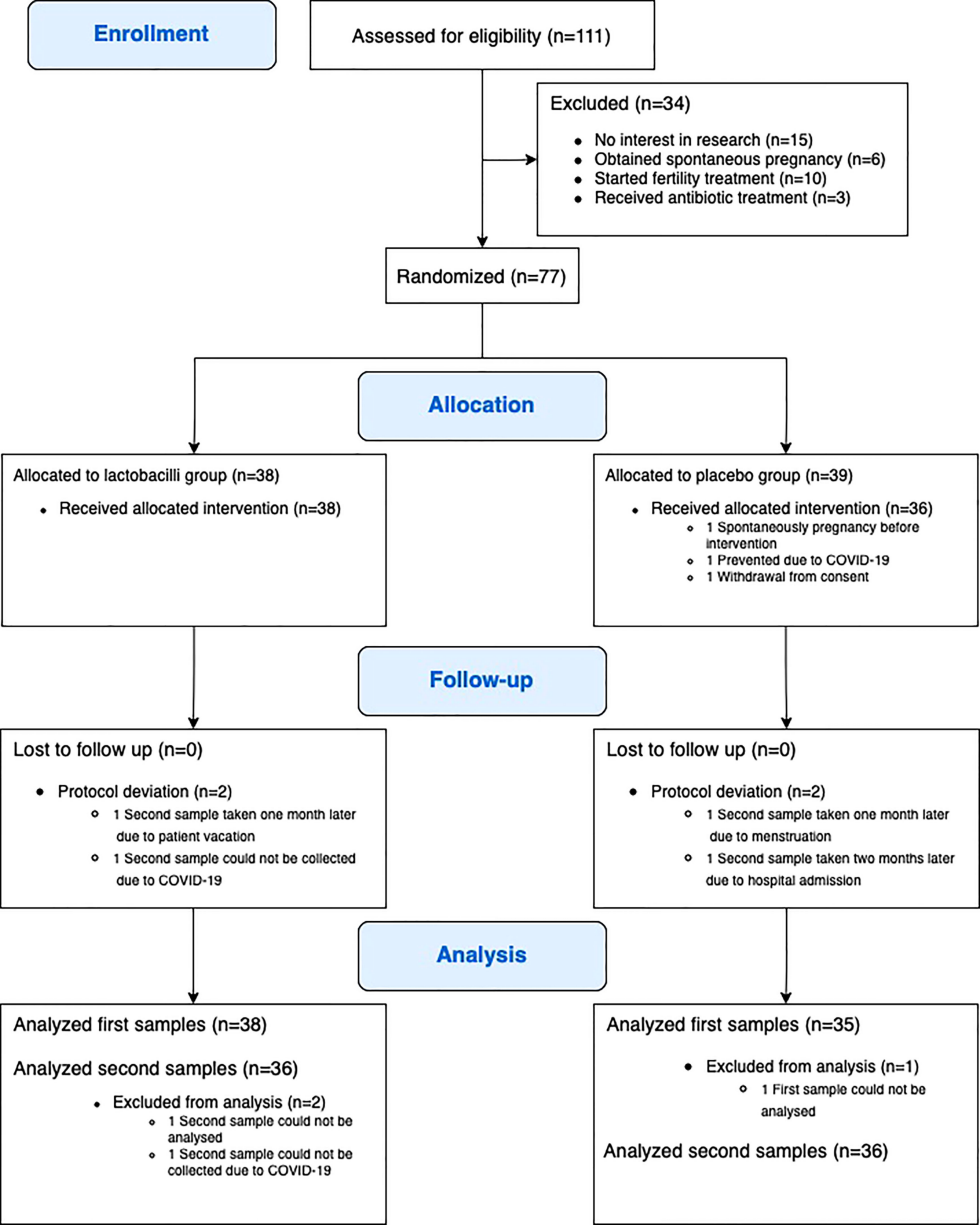
Flowchart of enrolment, allocation, follow-up, and analysis.

Between April 2019 and February 2021, a total of 77 participants were randomly assigned to either lactobacillus treatment or placebo (**Figure 1**). Three participants were excluded after randomization, one due to spontaneous pregnancy before the intervention, one due to COVID-19, and one participant withdrew consent before finishing intervention. Accordingly, 74 participants (38 participants in the lactobacillus group and 36 participants in the placebo group) completed the study intervention and follow-up. No adverse reactions were recorded.

**Table 1** presents the demographic and baseline characteristics of the participants. The mean age was 31 years (range 19–39). The mean BMI (kg/m^2^) was 26.4 (SD 5.6, range 18.3–39.9). The majority of the participants were Caucasian (97.3%). The two groups were comparable and well-balanced overall, except for the cause of infertility, where there was a higher proportion of male factor infertility and a lower proportion of idiopathic infertility in the lactobacilli group compared to the placebo group. Three participants started an IVF cycle between the first and second samples (one in the lactobacilli group and two in the placebo group).

**Table 1:** Demographic and baseline characteristics of participants.

	Lactobacilli group	Placebo group	Total
	(n = 38)	(n = 36)	(n = 74)
Age (y), mean (SD)	30.6 (4.0)	31.5 (4.5)	31.0 (4.2)
BMI (kg/m^2^), mean (SD)	26.8 (6.1)	26.1 (5.2)	26.4 (5.6)
Smoker, n (%)	–	–	0 (0)
Partner status			
- Male partner, n (%)	30 (78.9)	29 (80.6)	59 (79.7)
- Female partner, n (%)	2 (5.3)	0 (0)	2 (2.7)
- No partner, n (%)	6 (15.8)	7 (19.4)	13 (17.6)
- Other/no answer, n (%)	–	–	0 (0)
Ethnicity			
- Caucasian, n (%)	37 (97.4)	35 (97.2)	72 (97.3)
- Mediterranean, n (%)	1 (2.6)	1 (2.8)	2 (2.7)
- Other, n (%)			0 (0)
Cause of infertility			
- Idiopathic, n (%)	8 (21.1)	15 (41.7)	23 (31.1)
- Male factor, n (%)	18 (47.4)	9 (25.0)	27 (36.5)
- Tubar factor, n (%)	–	–	0 (0)
- Cycle disorder, n (%)	1 (2.6)	1 (2.8)	2 (2.7)
- Uterine factor, n (%)	–	–	0 (0)
- Combination, n (%)	2 (5.3)	1 (2.8)	3 (4.1)
- Other, n (%)	1 (2.6)	1 (2.8)	2 (2.7)
- No partner/female partner, n (%)	7 (18.4)	7 (19.4)	14 (18.9)
- Missing, n (%)	1 (2.6)	2 (5.6)	3 (4.1)
Penile-vaginal intercourse			
- None, n (%)	5 (13.2)	6 (16.7)	11 (14.9)
- Yes, 1–4 per month, n (%)	12 (31.6)	13 (36.1)	25 (33.8)
- Yes, 5–12 per month, n (%)	17 (44.7)	11 (30.6)	28 (37.8)
- Yes, >12 per month, n (%)	3 (7.9)	6 (16.7)	9 (12.2)
- Yes, frequency n.a. (%)	1 (2.6)	0 (0)	1 (1.4)
Self-assessed vaginal discharge			
- Normal, n (%)	26 (68.4)	31 (86.1)	57 (77.0)
- Increased, n (%)	11 (28.9)	5 (13.9)	16 (21.6)
- Missing, n (%)	1 (2.6)	0 (0)	1 (1.4)
Vaginal odor complaints			
- No, n (%)	31 (81.6)	25 (69.4)	56 (75.7)
- Yes, n (%)	6 (15.8)	11 (30.6)	17 (23.0)
- Missing, n (%)	1 (2.6)	0 (0)	1 (1.4)
Baseline vaginal microbiota profile			
- Low, n (%)	27 (71.1)	29 (80.6)	56 (75.7)
- Medium, n (%)	11 (28.9)	7 (19.4)	18 (24.3)

### Primary and secondary outcomes

#### Baseline to first sample

The microbiota profile was improved in 25 participants (34.2%) at the first sample after the intervention; 11 (28.9%) in the lactobacilli group and 14 (40.0%) in the placebo group (**Table 2**). There was no difference in improvement of the microbiota profile between the lactobacilli group and placebo group (primary outcome) (RR = 0.72 (95% CI 0.38–1.38), *P* = 0.32).

**Table 2:** Primary and secondary outcome: Improvement in the vaginal microbiota after intervention.

Primary outcome: Baseline to first sample	Improvement	No improvement	Total	Relative risk	95% CI	*P*-value
Lactobacilli group, n (% of group)	11 (28.9)	27 (71.1)	38 (100)	0.72	0.38–1.38	0.32
Placebo group, n (% of group)	14 (40.0)	21 (60.0)	35* (100)
Total, n (% of all)	25 (34.2)	48 (65.8)	73 (100)			
**Secondary outcome: Baseline to second sample**	**Improvement**	**No improvement**	**Total**	**Relative risk**	**95% CI**	***P*-value**
Lactobacilli group, n (% of group)	11 (30.6)	25 (69.4)	36** (100)	0.92	0.47–1.80	0.80
Placebo group, n (% of group)	12 (33.3)	24 (66.7)	36 (100)
Total, n (% of all)	23 (31.9)	49 (68.1)	72 (100)			

Improvement in the vaginal microbiota was defined as a change in the microbiota profile from low to medium, low to high, or medium to high from the baseline sample to the first sample (primary outcome) or from the baseline sample to the second sample (secondary outcome), whereas no improvement was defined as no change or deterioration.

*One sample could not be analyzed.

**One sample could not be analyzed, and one sample could not be collected due to COVID-19.

**Figure 2:**
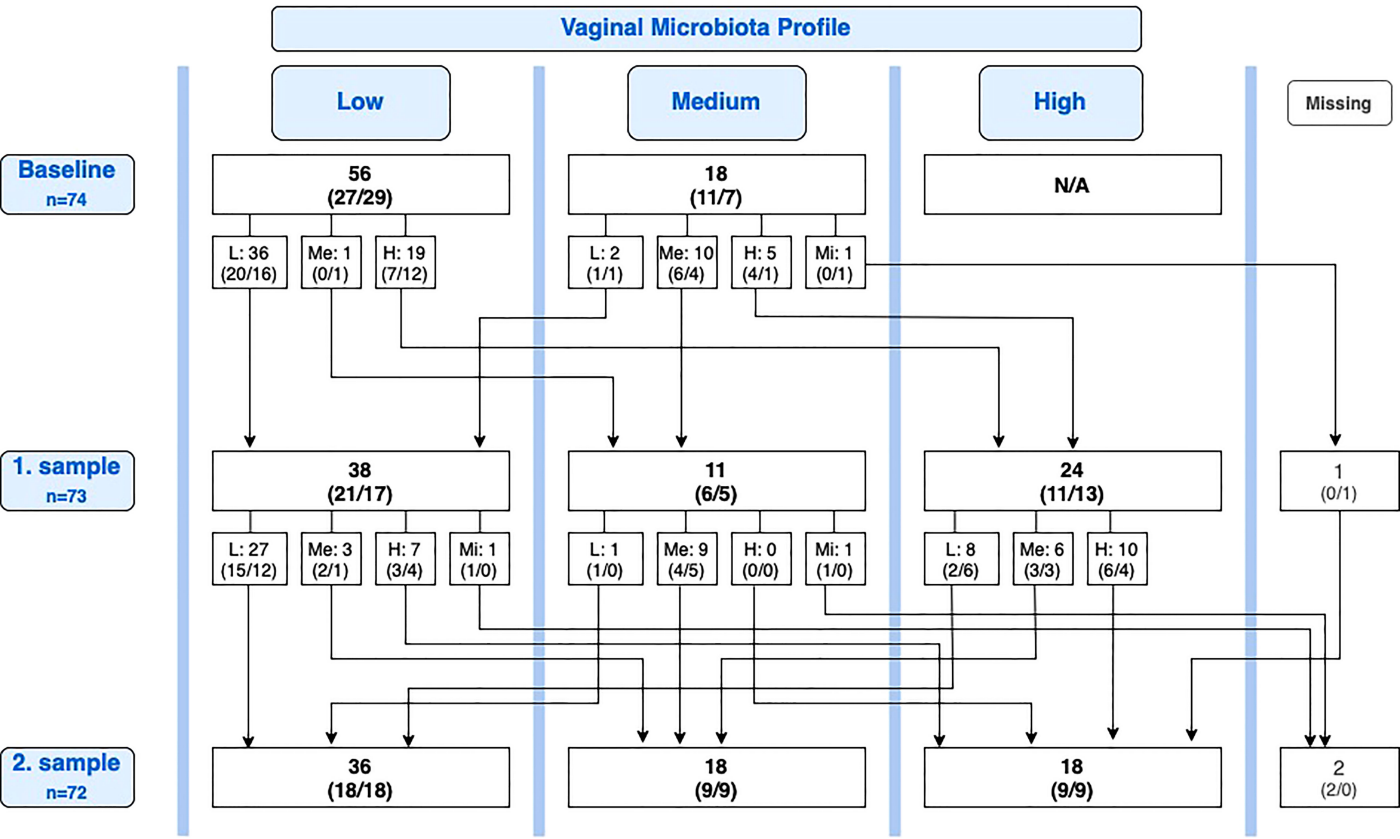
Overview of conversions in the vaginal microbiota between baseline, first sample, and second sample. Number of participants, n (lactobacilli-loaded capsules group, placebo group). L, Low; Me, Medium; H, High; Mi, Missing; N/A, Not applicable.

At baseline, 18 of 74 participants had a medium profile. Of these, two participants (11.1%) had deteriorated to low profile at the first sample, one in the lactobacilli group and one in the placebo group (**Figure 2**).

#### Baseline to second sample

In the cycle after the intervention cycle, the microbiota profile was improved compared with baseline in 23 participants (31.9%), 11 (30.6%) in the lactobacilli group, and 12 (33.3%) in the placebo group. In three participants, the second sample was taken respectively one, one, and two months later than protocolled, due to unforeseen circumstances (one improvement and two no improvement). Similarly, there was no difference in improvement between the lactobacilli group and the placebo group after the second sample (secondary outcome) (RR = 0.92 (95% CI 0.47–1.80), *P* = 0.80).

#### First to second sample

Of the 25 participants with improvement from baseline to first sample, 11 (44.0%) maintained an improved vaginal microbiota profile at the second sample, six in the lactobacilli group and five in the placebo group, while 14 (56.0%) deteriorated again. Reversely, of the 23 participants with improvement from baseline to second sample, eight (34.8%) were new improvements after the first sample. Of all 35 participants with a medium or high profile at the first sample, 15 (42.9%) had deteriorated to a lower profile at the second sample, six in the lactobacilli group and nine in the placebo group (*P* = 0.46; **Figure 2**).

### *Post-hoc* analyses

#### Spontaneous conversion rate

For participants overall, we performed a *post hoc* analysis of the spontaneous conversion rate (both deteriorations and improvements). **Figure 2** provides an overview of conversions from the baseline sample to the first sample and from the first sample to the second sample. Looking solely at the change in profile from the first sample to the second sample, equal in duration to one menstrual cycle, a one-cycle conversion rate of 35.2% (25 of 71 participants) was observed. Nearly all conversions were observed in the groups of women with low or high profiles (**Table 3**). Only one participant of 10 with a medium profile converted (to the less favorable low profile).

**Table 3:** Spontaneous conversions in the vaginal microbiota during one menstrual cycle for all participants.

First sample result	Second sample result			Total
	Deterioration	No change	Improvement	
Low, n (% of group)	n.a.	27 (73.0)	10 (27.0)	37 (100)
Medium, n (% of group)	1 (10.0)	9 (90.0)	0 (0)	10 (100)
High, n (% of group)	14 (58.3)	10 (41.7)	n.a.	24 (100)
Total, n (% of all)	15 (21.1)	46 (64.8)	10 (14.1)	71 (100)

n.a., not applicable.

#### Microbiota composition

Baseline samples revealed near identical compositions of the vaginal microbiota in the two groups before and after intervention. There was no difference in the abundance of all *Lactobacilli* between the groups neither before intervention (baseline: lactobacilli group 47.9% vs. placebo group 45.9%, *P* = 0.81) nor after intervention (first sample: lactobacilli group 51.4% vs. placebo group 47.9%, *P* = 0.75).

#### Spontaneous pregnancy during the study period

Five participants became spontaneously pregnant in the study period between intervention and the second sample, one in the lactobacilli group and four in the placebo group, 2.6% vs. 11.1% (*P* = 0.19).

#### Sexual activity

Overall, 11 participants (14.9%) reported not engaging in penile-vaginal intercourse. The improvement rate in this group of women was not different from the group of women engaging in penile-vaginal intercourse, 40.0% vs. 33.8% (*P* = 0.71).

## Discussion

### Summary of the main findings

This double-blinded, placebo-controlled randomized study showed no significant effect of treatment with lactobacilli-loaded vaginal capsules (> 10^8^ CFU of *Lactobacillus gasseri* EB01 DSM14869 and > 10^8^ CFU *Lactobacillus rhamnosus* PB01 DSM14870) on the unfavorable vaginal microbiota profile, defined as low or medium profiles by the IS-pro™ diagnostic system, among women referred to fertility treatment. Therefore, the results do not support the use of this specific probiotic vaginal capsule to improve the vaginal microbiota before fertility treatment. However, this study reveals the highly dynamic nature of the vaginal microbiota, with a spontaneous improvement rate of 34.2% being observed one to three months after the baseline sample. Over the course of one menstrual cycle, the spontaneous conversion rate of the vaginal microbiota (both deteriorations and improvements) was 35.2%. The medium profile appeared to be relatively more stable than low and high profiles.

These findings appear to be at odds with the beneficial effects of vaginal probiotic supplements in addition to antibiotics in symptomatic bacterial vaginosis (32). However, bacterial vaginosis represents a clinically symptomatic pathology caused by severe vaginal dysbiosis, whereas the vaginal microbiota deemed unfavorable for implantation appears to be an asymptomatic variant of the normal vaginal microbiota, which would otherwise remain undetected. Hence, it is plausible that the microbiological effects of introducing specific lactobacilli in the vaginal milieu are different in the two cases. Our findings also contrast with a previous study (33) which showed that lactobacilli supplements containing *Lactobacillus rhamnosus* and *Lactobacillus fermentum* could restore healthy vaginal flora in up to 82% of women with previous vaginal dysbiosis. However, this study was again focused on treating clinical bacterial vaginosis as defined by the Nugent Score and did not apply next-generation sequencing technology. Finally, it is unclear how the presents results on the vaginal microbiota relate to treatment of the endometrial dysbiosis where a previous has suggested that probiotics in combination with antibiotics may be beneficial (34).

The negative findings reported in this study could be accounted for by a number of factors. First, the underlying premise that vaginal probiotic tablets can modulate non-pathological variants of the vaginal microbiota could be incorrect. Although suggested by several researchers (35, 36), a recent systematic review of probiotic therapy in couples with infertility concluded that evidence is sparse and conflicting regarding the impact of probiotic treatment on clinical pregnancy rates and emphasized the need for randomized controlled trials (24). Molina et al. have recently suggested a number of other factors, both technical and individual, which may impact the microbiota to a greater extent than specific interventions (18).

Second, these findings might be accounted for by the lactobacilli formulation used in the present study. The formulation tested contained *Lactobacillus gasseri and Lactobacillus rhamnosus*. However, the analytic algorithm used to discern the division of profiles into low, medium, and high are not based on the relative abundance of these specific lactobacilli but rather the presence of lactobacilli in general and the relative presence of the specific *Lactobacillus crispatus* strain (17). Therefore, results could possibly be different were a different formulation of the lactobacilli loaded capsules to be used, e.g. one containing *Lactobacillus crispatus* (37).

Third, the results could reflect the validity of the IS-pro™ diagnostic system. However, the overall goal of research in probiotic therapy is to improve the chances of pregnancy following IVF treatment and since the IS-pro™ diagnostic system has been shown to be predictive of pregnancy chances (17), it was selected for use in this study. Other analytic methods exist but assuming a common underlying microbiological concept of favorability, it can be postulated that other analytic approaches proposed to correlate with pregnancy chances would produce similar results.

Fourth and finally, it may be that the impact of specific strains is dependent on the broader microbiota system and that multiple rather than single strain interventions are necessary to induce a clinically significant change.

### Limitations

Despite the careful design of this randomized controlled study, some limitations must be addressed. This study was carried out at a single center in a patient cohort that was overwhelmingly Caucasian. Inclusion of a more diverse patient population would have produced more generally applicable data but would have required a larger study population as the vaginal microbiota, including the dominance of *Lactobacillus* spp., varies among women of different ethnicities (9, 38).

It should also be noted that the intervention and placebo were self-administered. However, all patients were carefully instructed, and reviewing of excess medicine returned did not indicate compliance issues.

The IS-pro technique is based on a prediction algorithm evaluating specific taxa, but it does not consider the complete microbiota or the presence of specific pathogenic bacteria. Moreover, it does not consider the endometrial microbiota which also is suggested to impact the reproductive outcome of IVF (39).

The time from the baseline screening sample to the first sample was designed to range from 1–3 months. Within this timeframe, spontaneous improvement may have occurred in some patients before the intervention was administered. However, this time frame was selected based on the prognostic value of the IS-pro analysis system on pregnancy rates found by Koedooder et al. (17) and the randomized controlled design of the study eliminates the risk of bias from this effect.

Finally, we defined both low and medium profiles as unfavorable. This could have decreased the observed improvement rates due to the relatively higher stability of the medium profile. However, patients with a medium profile were included since the potential near two-fold increase in pregnancy rates found by Koedooder et al. (23.8% vs. 52.6% in patients with a high profile) would be highly clinically relevant.

### Clinical implications

The present study provides two novel insights into the clinical application of vaginal microbiota profile assessment in women undergoing fertility treatment. First, the results provide no evidence to support the use of lactobacilli-loaded capsules containing *L. gasseri* and *L. rhamnosus* to improve the vaginal microbiota before fertility treatment. While not beneficial, the study did not indicate any adverse effects of this probiotic therapy. Second, the study confirmed the dynamic nature of the vaginal microbiota and found that spontaneous improvement of an unfavorable vaginal microbiota is likely to occur in 34.2% of patients with an unfavorable microbiota within a period of one to three months. This may suggest that a strategy of postponing further IVF treatment to await microbiota improvement could be helpful in some patients as previously suggested (17). However more evidence for the efficacy of postponing IVF/ICSI treatment to improve the microbiota is required before it can be recommended as alternative strategy to probiotic treatment.

In conclusion, this study indicates that the ‘rush to probiotics’ should be tempered with some caution. More studies of both the vaginal and endometrial microbiota are required to confirm the efficacy of specific vaginal probiotics before they can be considered as a therapeutic solution in this context.

## Data availability statement

The raw data supporting the conclusions of this article will be made available by the authors, without undue reservation.

## Ethics statement

The study was approved by the Danish National Committee on Health Research Ethics (SJ-710). The patients/participants provided their written informed consent to participate in this study.

## Author contributions

MS, TH, and NM designed and conceived the trial. IE, MS, NM, and AE planned the statistical analyses. IE gathered and analyzed data and drafted the initial manuscript. MS, AE, KP, and MW assisted in data collection and interpretation. All authors have critically reviewed the manuscript and approved the final version.

## Funding

This study was unconditionally funded by ReproHealth Research Consortium Zealand University Hospital, and Savvaerksejer Jeppe Juhls og Hustrus Ovita Juhls Fund. Deerland Probiotics & Enzymes A/S unconditionally provided the vaginal supplements Vivag Plus^®^ and the placebo supplements. The company did not have any role in the design or conduct of the study or the preparation of the manuscript. ARTPred provided the ReceptIVFity tests at a study discounted rate. The company did not have any role in the design or conduct of the study or the preparation of the manuscript.

## Acknowledgments

The authors wish to thank and acknowledge the statistics department, OUH OPEN Statistics, Odense University Hospital, and Department of Clinical Research, University of Southern Denmark, for statistical guidance. We also thank our co-worker Line Buur Dessing, research and project nurse at the Department of Gynecology, Obstetrics, and Fertility, Zealand University Hospital, Denmark. Last, we also thank ARTPred, Amsterdam, the Netherlands, for constructive cooperation.

## Conflict of interest

MW reports a personal fee from Merck for a lecture. NM reports consultancy fees from ARTPred.

The remaining authors declare that the research was conducted in the absence of any commercial or financial relationships that could be construed as a potential conflict of interest.

## Publisher’s note

All claims expressed in this article are solely those of the authors and do not necessarily represent those of their affiliated organizations, or those of the publisher, the editors and the reviewers. Any product that may be evaluated in this article, or claim that may be made by its manufacturer, is not guaranteed or endorsed by the publisher.

## References

[1] Rahmati M Macklon N. Testing the endometrium: Is there enough evidence to justify clinical use? Curr Opin Obstet Gynecol (2020) 32:185–90. doi: 10.1097/GCO.0000000000000627 32251094

[2] García-Velasco JA Menabrito M Catalán IB. What fertility specialists should know about the vaginal microbiome: a review. Reprod BioMed Online (2017) 35:103–12. doi: 10.1016/j.rbmo.2017.04.005 28479120

[3] Bracewell-Milnes T Saso S Nikolaou D Norman-Taylor J Johnson M Thum MY. Investigating the effect of an abnormal cervico-vaginal and endometrial microbiome on assisted reproductive technologies: A systematic review. Am J Reprod Immunol (2018) 80. doi: 10.1111/aji.13037 30133062

[4] Singer M Borg M Ouburg S Morré SA. The relation of the vaginal microbiota to early pregnancy development during *in vitro* fertilization treatment–a meta-analysis. J Gynecol Obstet Hum Reprod (2019) 48:223–9. doi: 10.1016/j.jogoh.2019.01.007 30685426

[5] Marchesi JR Ravel J. The vocabulary of microbiome research: a proposal. Microbiome (2015) 3:1–3. doi: 10.1186/s40168-015-0094-5 26229597 PMC4520061

[6] Huttenhower C Gevers D Knight R Abubucker S Badger JH Chinwalla AT. Structure, function and diversity of the healthy human microbiome. Nature (2012) 486:207–14. doi: 10.1038/nature11234 PMC3564958 22699609

[7] Yamamoto T Zhou X Williams CJ Hochwalt A Forney LJ. Bacterial populations in the vaginas of healthy adolescent women. J Pediatr Adolesc Gynecol (2009) 22:11–8. doi: 10.1016/j.jpag.2008.01.073 19232297

[8] Gajer P Brotman RM Bai G Sakamoto J Schütte UME Zhong X. Temporal dynamics of the human vaginal microbiota. Sci Transl Med (2012) 4:132ra52. doi: 10.1126/scitranslmed.3003605 PMC3722878 22553250

[9] Ravel J Gajer P Abdo Z Schneider GM Koenig SSK McCulle SL. Vaginal microbiome of reproductive-age women. Proc Natl Acad Sci U.S.A. (2011) 108:4680–7. doi: 10.1073/pnas.1002611107 PMC3063603 20534435

[10] Hay PE Lamont RF Taylor-Robinson D Morgan DJ Ison C Pearson J. Abnormal bacterial colonisation of the genital tract and subsequent preterm delivery and late miscarriage. Bmj (1994) 308:295. doi: 10.1136/bmj.308.6924.295 8124116 PMC2539287

[11] Hillier SL Nugent RP Eschenbach DA Krohn MA Gibbs R Martin DH. “Association between bacterial vaginosis and preterm delivery of a low- birth-weight infant. Stud Fam Plann (1996) 27:57. doi: 10.2307/2138082 7491137

[12] Donders GG Van Calsteren K Bellen G Reybrouck R Van Den Bosch T Riphagen I. Predictive value for preterm birth of abnormal vaginal flora, bacterial vaginosis and aerobic vaginitis during the first trimester of pregnancy. BJOG (2009) 116:1315–24. doi: 10.1111/j.1471-0528.2009.02237.x 19538417

[13] Brown RG Al-Memar M Marchesi JR Lee YS Smith A Chan D. Establishment of vaginal microbiota composition in early pregnancy and its association with subsequent preterm prelabor rupture of the fetal membranes. Trans Res (2019) 207:30–43. doi: 10.1016/j.trsl.2018.12.005 PMC6489901 30633889

[14] Hyman RW Herndon CN Jiang H Palm C Fukushima M Bernstein D. The dynamics of the vaginal microbiome during infertility therapy with *in vitro* fertilization-embryo transfer. J Assist Reprod Genet (2012) 29:105–15. doi: 10.1007/s10815-011-9694-6 PMC3270134 22222853

[15] Haahr T Jensen JS Thomsen L Duus L Rygaard K Humaidan P. Abnormal vaginal microbiota may be associated with poor reproductive outcomes: A prospective study in IVF patients. Hum Reprod (2016) 31:795–803. doi: 10.1093/humrep/dew026 26911864

[16] Bernabeu A Lledo B Díaz MC Lozano FM Ruiz V Fuentes A. Effect of the vaginal microbiome on the pregnancy rate in women receiving assisted reproductive treatment. J Assist Reprod Genet (2019) 36:2111–9. doi: 10.1007/s10815-019-01564-0 PMC6823330 31446545

[17] Koedooder R Singer M Schoenmakers S Savelkoul PHM Morré SA de Jonge JD. The vaginal microbiome as a predictor for outcome of *in vitro* fertilization with or without intracytoplasmic sperm injection: a prospective study. Hum Reprod (2019) 34:1042–54. doi: 10.1093/humrep/dez065 31119299

[18] Molina NM Sola-Leyva A Jose Saez-Lara M Plaza-Diaz J Tubic-Pavlovic A Romero B. New opportunities for endometrial health by modifying uterine microbial composition: Present or future? Biomolecules (2020) 10:593. doi: 10.3390/biom10040593 PMC7226034 32290428

[19] García-Velasco JA Budding D Campe H Malfertheiner SF Hamamah S Santjohanser C. The reproductive microbiome – clinical practice recommendations for fertility specialists. Reprod BioMed Online (2020) 41:443–53. doi: 10.1016/j.rbmo.2020.06.014 32753361

[20] Hemalatha R Mastromarino P Ramalaxmi BA Balakrishna NV Sesikeran B. Effectiveness of vaginal tablets containing lactobacilli versus pH tablets on vaginal health and inflammatory cytokines: A randomized, double-blind study. Eur J Clin Microbiol Infect Dis (2012) 31:3097–105. doi: 10.1007/s10096-012-1671-1 22777592

[21] Homayouni A Bastani P Ziyadi S Mohammad-Alizadeh-Charandabi S Ghalibaf M Mortazavian AM. Effects of probiotics on the recurrence of bacterial vaginosis: A review. J Low Genit Tract Dis (2014) 18:79–86. doi: 10.1097/LGT.0b013e31829156ec 24299970

[22] SenokACVerstraelenHTemmermanMBottaGA. Probiotics for the treatment of bacterial vaginosis. Cochrane Database Syst Rev (2009), 4. doi: 10.1002/14651858.CD006289.PUB2/MEDIA/CDSR/CD006289/IMAGE_N/NCD006289-AFIG-FIG02.PNG 19821358

[23] Wang Z He Y Zheng Y. Probiotics for the treatment of bacterial vaginosis: A meta-analysis. Int J Environ Res Public Health (2019) 16:3859. doi: 10.3390/ijerph16203859 PMC6848925 31614736

[24] Corbett GA Crosby DA McAuliffe FM. Probiotic therapy in couples with infertility: A systematic review. Eur J Obstetr Gynecol Reprod Biol (2021) 256:95–100. doi: 10.1016/j.ejogrb.2020.10.054 33188995

[25] Schulz KF Altman DG Moher D. CONSORT 2010 statement: Updated guidelines for reporting parallel group randomised trials. Int J Surg (2011) 9:672–7. doi: 10.1016/j.ijsu.2011.09.004 22019563

[26] Budding AE Hoogewerf M Vandenbroucke-Grauls CMJE Savelkoul PHM. Automated broad-range molecular detection of bacteria in clinical samples. J Clin Microbiol (2016) 54:934–43. doi: 10.1128/JCM.02886-15 PMC4809945 26763956

[27] Budding AE Grasman ME Eck A Bogaards JA Vandenbroucke-Grauls CMJE van Bodegraven AA. Rectal swabs for analysis of the intestinal microbiota. PloS One (2014) 9:e101344. doi: 10.1371/journal.pone.0101344 PMC4096398 25020051

[28] Daniels L Budding AE de Korte N Eck A Bogaards JA Stockmann HB. Fecal microbiome analysis as a diagnostic test for diverticulitis. Eur J Clin Microbiol Infect Dis (2014) 33:11 2014. doi: 10.1007/S10096-014-2162-3 24894339

[29] Eck A de Groot EFJ de Meij TGJ Welling M Savelkoul PHM Budding AE. Robust microbiota-based diagnostics for inflammatory bowel disease. J Clin Microbiol (2017) 55:1720. doi: 10.1128/JCM.00162-17 28330889 PMC5442528

[30] Eck A Zintgraf LM de Groot EFJ de Meij TGJ Cohen TS Savelkoul PHM. Interpretation of microbiota-based diagnostics by explaining individual classifier decisions. BMC Bioinf (2017) 18:313. doi: 10.1186/s12859-017-1843-1 PMC5628491 28978318

[31] Budding AE Grasman ME Lin F Bogaards JA Soeltan-Kaersenhout DJ Vandenbroucke-Grauls CMJE. IS-pro: high-throughput molecular fingerprinting of the intestinal microbiota. FASEB J (2010) 24:4556–64. doi: 10.1096/FJ.10-156190 20643909

[32] van de Wijgert JHHM Verwijs MC. Lactobacilli-containing vaginal probiotics to cure or prevent bacterial or fungal vaginal dysbiosis: a systematic review and recommendations for future trial designs. BJOG (2020) 127:287–99. doi: 10.1111/1471-0528.15870 31299136

[33] Reid G Beuerman D Heinemann C Bruce AW. Probiotic lactobacillus dose required to restore and maintain a normal vaginal flora. FEMS Immunol Med Microbiol (2001) 32:37–41. doi: 10.1111/j.1574-695x.2001.tb00531.x 11750220

[34] Kadogami D Nakaoka Y Morimoto Y. Use of a vaginal probiotic suppository and antibiotics to influence the composition of the endometrial microbiota. Reprod Biol (2020) 20:307–14. doi: 10.1016/j.repbio.2020.07.001 32680750

[35] Chenoll E Moreno I Sánchez M Garcia-Grau I Silva Á González-Monfort M. Selection of new probiotics for endometrial health. Front Cell Infect Microbiol (2019) 9:114. doi: 10.3389/fcimb.2019.00114 31058101 PMC6481279

[36] López-Moreno A Aguilera M. Probiotics dietary supplementation for modulating endocrine and fertility microbiota dysbiosis. Nutrients (2020) 12:1–15. doi: 10.3390/nu12030757 PMC7146451 32182980

[37] Lamont RF Sobel JD Akins RA Hassan SS Chaiworapongsa T Kusanovic JP. The vaginal microbiome: New information about genital tract flora using molecular based techniques. BJOG (2011) 118:533–49. doi: 10.1111/j.1471-0528.2010.02840.x PMC3055920 21251190

[38] Serrano MG Parikh HI Brooks JP Edwards DJ Arodz TJ Edupuganti L. Racioethnic diversity in the dynamics of the vaginal microbiome during pregnancy. Nat Med (2019) 25:1001. doi: 10.1038/S41591-019-0465-8 31142850 PMC6746180

[39] Moreno I Codoñer FM Vilella F Valbuena D Martinez-Blanch JF Jimenez-Almazán J. Evidence that the endometrial microbiota has an effect on implantation success or failure. Am J Obstet Gynecol (2016) 215:684–703. doi: 10.1016/j.ajog.2016.09.075 27717732

